# Draft Genome Sequence of the 1,2-Dichloroethane-Utilizing *Micrococcus* sp. Strain NDB3Y10, Isolated from an Australian Bore Well Producing Coal Seam Gas

**DOI:** 10.1128/genomeA.00255-17

**Published:** 2017-04-27

**Authors:** Joseph Adelskov, Bharat K. C. Patel

**Affiliations:** a School of Natural Sciences, Griffith University, Brisbane, Queensland, Australia; b School of Earth, Environment and Biological Sciences, Queensland University of Technology, Brisbane, Queensland, Australia

## Abstract

*Micrococcus luteus* strain NDB3Y10, which utilizes 1,2-dichloroethane as a carbon source, was isolated from a bore well that produces coal seam gas. The draft genome size of the strain was 2.49 Mb with a G+C content of 72.97%. Genes involved in the metabolism of halogenated substrates, including halogenated hydrocarbons, were identified.

## GENOME ANNOUNCEMENT

The genus *Micrococcus*, a member of the phylum *Actinobacteria*, currently consists of 17 taxonomically valid species (http://www.bacterio.net). Members of the genus *Micrococcus* are found in a wide range of diverse environments, including the type species *Micrococcus luteus*, a known commensal that is found in humans and can contribute to food spoilage (1). *Micrococcus* sp. strain NDB3Y10 was isolated from a water sample collected from a newly drilled (65 h) gas bore well located in the upper Surat Basin, Roma, Queensland, Australia. Strain NDB3Y10 grew optimally under aerobic conditions at 37°C and pH 8 in basal PL medium (2) supplemented with 0.2% yeast extract, glucose, and tryptone. The strain was also able to grow on PL medium supplemented with 0.01% yeast extract and 10 mM 1,2-dichloroethane (1,2-DCE), a highly toxic environmental pollutant. Partial 16S rRNA gene sequencing analysis revealed that *Micrococcus luteus* DSM 20030^T^ was the nearest phylogenetic neighbor with a sequence identity of 96.44%. Further analysis using Biolog plates (GN2 Micro Plate, Biolog, USA) showed that the strain metabolized several different carboxylic acids, saccharides, pyruvic acid methyl ester, and succinic acid mono-methyl-ester. We report here the first genome sequence of the 1,2-DCE-utilizing *Micrococcus* sp. strain NDB3Y10, isolated from a water sample collected from a newly drilled bore well producing coal seam gas.

High-molecular-weight DNA was extracted from a late log phase culture of strain NDB3Y10 using a modification of Marmur’s method (3). The DNA was sent to the Australian Genome Research Facility (AGRF) for TruSeq library prep and sequencing on the Illumina MiSeq platform. The sequencing run produced a total of 1,306,503 paired-end reads (250 bp), which were reduced to 1,296,434 reads after quality filtering. *De novo* assembly was performed using SPAdes version 3.5.0, which produced a high-quality draft genome consisting of 84 contigs (≥500 bp in length) with an *N*_50_ of 64,137 bp and an average genome coverage of 247×. The contigs had a G+C content of 72.97% and a combined length of 2,497,512 bp, which is estimated to cover 99.85% of the total genome length.

Annotation of the draft genome of strain NDB3Y10 using Prokka version 1.12b (4) revealed the presence of 2,274 protein-coding and 55 RNA gene sequences. Average nucleotide identity using BLAST (ANIb) analysis showed that strain NDB3Y10 shared the highest ANIb value (>98%) with *M. luteus* strains SK58 and 773_MLUT, which were isolated from human hosts. A comparative analysis using Roary (5) identified genes involved in the uptake of molybdenum and in the biosynthesis of molybdopterin, as well as a methane monoxygenase in strain NDB3Y10, thus excluding the other 11 genomes of *M. luteus* strains (SK85, modasa, NCTC 2665, trpE16, RIT304, RIT305, RIT324W, SUBG006, 773_MLUT, 1058_MLUT, and SEN31). RAST (6) annotations of the genome of strain NDB3Y10 identified haloacid dehalogenase and haloalkane dehydrogenase-like genes, a class of hydrolytic dehalogenases involved in the degradation of halogenated hydrocarbons such as 1, 2-DCE (7). This constitutes the first report of the 1,2-DCE-utilizing *Micrococcus* sp. strain NDB3Y10 in which potential hydrolytic dehalogenases have been identified.

### Accession number(s).

This whole-genome shotgun project has been deposited in GenBank under the accession number LQAC00000000. The version described in this paper is the first version, LQAC00000000.1.
